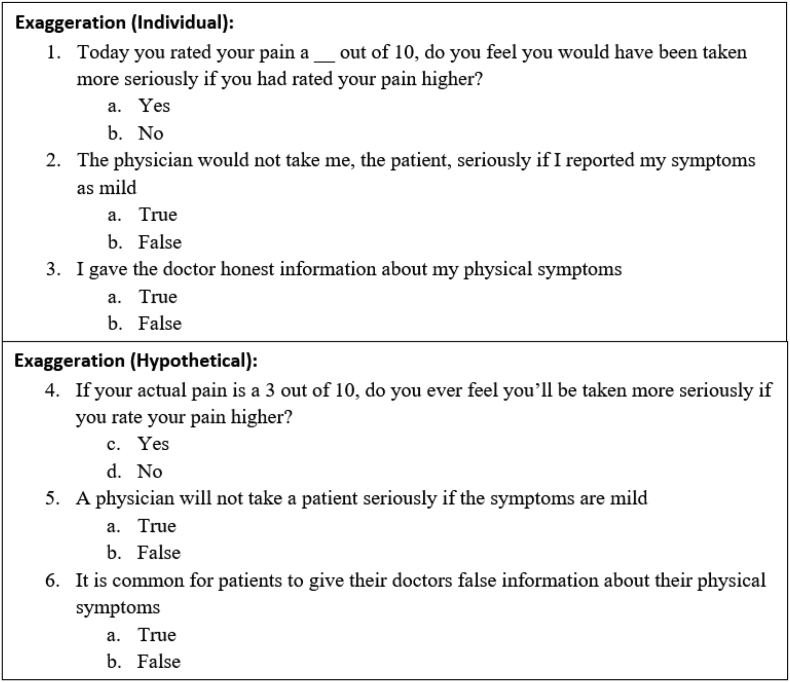# Patient symptom exaggeration is associated with communication effectiveness and trust^☆^

**DOI:** 10.1016/j.pecinn.2022.100050

**Published:** 2022-05-10

**Authors:** Faiza Sarwar, Tom Crijns, Sina Ramtin, David Ring, Lee Reichel, Amirreza Fatehi

**Affiliations:** Department of Orthopedic Surgery, Dell Medical School, The University of Texas at Austin, Austin, Texas, USA

**Keywords:** Symptom exaggeration, Communication effectiveness, Trust, Patient-physician communication, Symptom magnification

## Abstract

**Objective:**

Patients might exaggerate their symptoms in an attempt to align the clinician’s views with their own. A person who sees potential benefit in symptom exaggeration might also experience less trust, more difficulty communicating, and lower satisfaction with their clinician. We asked if there was an association between patient rating of communication effectiveness, patient satisfaction, and patient trust with symptom exaggeration?

**Methods:**

One hundred and thirty-two patients in four orthopaedic offices completed surveys including demographics, Communication-Effectiveness-Questionnaire (CEQ-6), Negative-Pain-Thoughts-Questionnaire (NPTQ-4), a Guttman-style satisfaction question, PROMIS Depression, and Stanford Trust in Physician. Patients were randomly assigned to answer three questions about symptom exaggeration for two scenarios: 1) their own exaggeration during the just-completed visit or 2) the average person’s tendency to exaggerate.

**Results:**

In multivariable analysis, lower ratings of communication effectiveness were associated with greater symptom exaggeration (p=0.002), while an annual household income>$100,000 (p=0.033) was associated with higher ratings. Higher rating of satisfaction was associated with lower education attained (p=0.004). Greater trust was associated with lower personal exaggeration (p=0.002).

**Conclusion:**

The relationship between greater exaggeration and lower ratings of communication effectiveness and trust suggests that symptom descriptions that seem more intense or diffuse than expected may indicate opportunities for more effective communication and trust.

**Innovation:**

Patient experience can be improved by training clinicians to identify symptom exaggeration as a signal that the patient does not feel heard and understood and a cue to return to communication strategies that build trust.

## Highlights

• Lower ratings of communication effectiveness and trust were associated with greater symptom exaggeration.
• Symptom descriptions that seem more intense than expected may indicate shortcomings in the patient-clinician relationship.
• Clinicians identifying symptom exaggeration as a signal that patients don't feel understood can improve patient experience.

## 1 Introduction

The number, distribution, and severity of symptoms often increases during the course of an office visit, a phenomenon sometimes referred to as symptom amplification or symptom exaggeration [1]. The concept of “exaggeration” intentionally posits that people may be using the intensity, distribution, and impact of symptoms—consciously or unconsciously--as a communication tactic. For instance, a bothersome hand or wrist symptom at the start of a visit can come to involve most of the upper limb and limit simple daily activities. It is possible that patients exaggerate their symptoms in an attempt to be taken seriously by the clinician [2].

There is evidence that both verbal and non-verbal aspects of symptom communication correlate with misinterpretation of symptoms and greater feelings of worry or despair [[3], [4], [5]]. For example, aspects of communication such as extreme words like “unbearable” or expression that the problem seems serious such as “I have a high threshold for pain” can be signs of unhelpful thinking (such as “painful activity means I’m making the problem worse”) [4]. The same is true for non-verbal communication, such as holding one’s upper extremity as if it’s detached or fragile, or bending the wrist when trying to make a fist [5].

In the same vein, descriptions of symptoms that become more diffuse, more intense, more more disabling as the interview progresses might represent a patient signaling that they do not feel heard and understood by the clinician. In other words, symptom exaggeration may be a sign that the patient-clinician relationship is deteriorating. It might serve as a prompt for clinicians to employ specific communication strategies for restoring trust and mutual respect [6,7].

Patients and clinicians often differ on their interpretation of symptoms and their weighting of test and treatment options [[8], [9], [10]]. It is possible that patients might exaggerate their symptoms in an attempt to align the clinician’s views with their own [2]. A person that sees potential benefit in symptom exaggeration might also experience less trust, more difficulty communicating, and lower satisfaction with their clinician. There is evidence that patients experience greater trust when they feel listened to, feel they receive the information they need, feel involved in decision making, and feel they spent as much time as they need with their physician [[11], [12], [13], [14], [15]]. And trust increases adherence and loyalty [[16], [17], [18], [19]]. Attitudes towards symptom experience are worthy of study, because perceived symptom exaggeration could be an important prompt for clinicians to employ specific communication strategies to focus on restoring trust and mutual respect [6,7].

This study addressed the following research questions: 1) Is there an association between patient rating of communication effectiveness and symptom exaggeration accounting for other factors? 2) Is there an association between patient satisfaction and symptom exaggeration? 3) Is there an association between trust in the clinician and symptom exaggeration?

## 2 Methods

### 2.1 Study design and setting

In this cross-sectional, institutional review board-approved, observational study, we enrolled 132 patients presenting to one of four orthopaedic offices in an urban area of the United States. Musculoskeletal illness largely addresses quality of life issues, tests and treatments tend to be discretionary and preference-sensitive, and misinterpretation of symptoms and distress regarding symptoms is commonplace. Consequently, musculoskeletal specialty care is a good setting for studying the biopsychosocial paradigm of illness in general and patient experience specifically. All new and return English speaking patients between the age of 18 to 88 were included. After verbal invitation and a recruitment letter, completing questionnaires implied consent.

### 2.2 Measurements

Participants completed the six-item Communication Effectiveness Questionnaire (CEQ-6), the brief Negative Pain Thoughts Questionnaire (NPTQ-4), a Guttman-style measure of satisfaction with care, the Patient-reported Outcomes Measurement Information System (PROMIS) Depression computerized adaptive test (CAT), the Stanford Trust in Physician Scale, and a basic demographics survey. In addition, patients were randomly assigned (simple randomization, 1:1) to answer three questions about symptom exaggeration for two scenarios: 1) their own exaggeration during the just-completed visit (ex: “Today you rated your pain a __ out of 10, do you feel you would have been taken more seriously if you had rated your pain higher? Yes or No”; Appendix D) or 2) the average person’s tendency to exaggerate (ex: "A physician will not take a patient seriously if the symptoms are mild: True or False?”; Appendix D). Data were recorded on the web-based, HIPAA-compliant Research Electronic Data Capture (REDCap) system.

The Communication Effectiveness Questionnaire consists of 6 items measuring the patient’s perspectives regarding clinician communication during the visit [20]. Reponses were rated on a 7-point Likert scale from strongly disagree to strongly agree. Total scores range from 6 to 42, with higher scores indicating more effective clinician communication as perceived by the patient, The Negative Pain Thoughts Questionnaire is a 4-item validated measure of common unhelpful thoughts in response to nociception [21]. Patients rated answers on a 6-point Likert scale from strongly disagree to strongly agree. Total scores range from 4 to 24, with higher scores indicating more negative thoughts associated with their pain. A Guttman-type (iterative) Satisfaction Scale is an iterative measure of patient satisfaction with care which has a lower ceiling effect in comparison to a Likert or ordinal satisfaction scale [22]. The questionnaire is designed to readjust based on the patient’s prior response. For example, the first question asks ‘Was today’s visit satisfactory?,’ and if the patient answered ‘yes,’ the next question would be ‘Was today’s visit more satisfactory than usual?’ (vs. ‘Was today’s visit more dissatisfactory than usual?’). The Patient-reported Outcomes Measurement Information System (PROMIS) Depression Computerized Adaptive Test is a valid measure of symptoms of depression with a score of 50 representing the U.S. population mean [23]. The Stanford Trust in Physician Scale is a validated 11-item self-reported measure of patient trust in their physician [24]. Each question is rated on a 5-point Likert scale from strongly disagree to strongly agree. We asked 3 questions about benefit in exaggerating symptoms. Each item was a yes/no or true/false question score as 0 or 1, with scores ranging from 0-3. Higher scores indicated greater agreement, indicating that the patient was more likely to consider exaggerating symptoms as a method of communication with their clinician. Symptom exaggeration was the primary response variable and we sought its association with communication effectiveness, satisfaction, and trust.

### 2.3 Participants

The mean age of the 132 participants was 47 years and 38% were men (Table 1).

**Table 1:** Patient demographics.

Variable	Value
N	132
Age	47 ± 16
Gender	
Woman	81 (61%)
Man	50 (38%)
Other	1 (0.76%)
Race/ethnicity	
White	72 (55%)
Hispanic	32 (24%)
Black or African American	15 (11%)
Other	13 (9.9%)
Marital status	
Single	42 (32%)
Married	64 (48%)
Divorced, separated, or widowed	26 (20%)
Level of education	
High school or lower	28 (21%)
2-year college	20 (15%)
4-year college	44 (33%)
Postgraduate degree	40 (30%)
Work status	
Employed	83 (63%)
Unemployed	13 (9.9%)
Disabled	7 (5.3%)
Other	29 (22%)
Annual household income	
Less than $15,000	23 (17%)
From $15,000 to $29,999	13 (9.9%)
From $30,000 to $49,999	19 (14%)
From $50,000 to $99,999	19 (14%)
More than $100,000	58 (44%)
Insurance status	
Medicaid	10 (7.6%)
Medicare	17 (13%)
Private	76 (58%)
None or other	29 (22%)
CEQ-6 (total)	38 ± 6.0
NPTQ-4 (total)	9 (5.5-12)
Guttman satisfaction	5.8 ± 1.3
PROMIS Depression (t-score)	50 ± 8.8
Trust in Physician scale (total)	37 (35-39)
Symptom exaggeration	
Hypothetical	
0	41 (62%)
1	19 (29%)
2	3 (4.6%)
3	3 (4.6%)
Individual	
0	56 (85%)
1	7 (11%)
2	3 (4.6%)
3	0 (0%)

Continuous variables as mean ± standard deviation or median (interquartile range); categorical variables as number (percentage). CEQ-6= Communication Effectiveness Questionnaire, 6-item; NPTQ-4= Negative Pain Thoughts Questionnaire, 4-item; PROMIS= Patient-Reported Outcomes Measurement Information System.

### 2.4 Statistical analysis

We performed descriptive statistics of all participants. Continuous variables were reported as mean ± standard deviation or median (interquartile range); categorical variables were reported as number (percentage). We sought factors associated with communication effectiveness, satisfaction, and trust in the clinician in bivariate analysis. Mann-Whitney U tests and Kruskal-Wallis H tests were used for categorical variables, while Spearman rank correlations were calculated for continuous variables. We planned parsimonious models to limit overfitting. If there was only one variable associated in bivariate analysis, a multivariable model would not be performed. All variables with *P* < 0.10 were moved to multivariable regression analysis. The extent of symptom exaggeration was pooled when patients had 2 or more items, and both patients randomized to the hypothetical and personal questions were analyzed in the same model. In addition, we separated patients by personal, hypothetical, and sought factors associated with the CEQ-6, Guttman satisfaction, and the Stanford Trust in Physician scale. Given the nonparametric distribution of the CEQ-6 and the Guttman satisfaction scale, we used Poisson regression to identify factors associated with each response variable. In order to indicate the magnitude of effect in the Poisson models, we calculated the delta-Akaike Information Criterion (AIC). The AIC of the full model was subtracted from the AIC of the model without the target variable. Lower AIC values indicate better fit, and therefore, higher delta-AIC values indicate that a larger proportion of variation in the response variable is explained by a given factor. Regression coefficients (RC), 95% Confidence Intervals (95% CI), standard errors, delta-AIC values, and *P-*values were reported.

An a priori sample size calculation demonstrated that 129 patients would provide 80% statistical power, based on a linear regression with if symptom exaggeration would account for 5% or more of the variability in communication effectiveness, and if the complete model would account for 20% or more variability.

## 3 Results

Accounting for potential confounding using multivariable analysis, lower ratings of communication effectiveness (CEQ-6) were associated with greater symptom exaggeration (checking ‘yes’ on two or more items; RC= -0.21; 95% CI= p=0.002), while an annual household income of greater than $100,000 (Regression Coefficient= 0.11, p=0.033) was associated with higher ratings (Table 3).

Higher satisfaction with care was associated with lower education alone in bivariate analysis (p=0.004) (Table 2). We did not perform multivariable analysis.

**Table 2:** Bivariate analysis of patient factors associated with communication effectiveness, satisfaction, and trust in the physician.

	CEQ-6		Satisfaction (Guttman scale)		Trust in Physician scale	
Categorical variables	Median (IQR)	*P*-value	Mean ± SD	*P*-value	Mean ± SD	*P*-value
Symptom exaggeration		0.16		0.61		0.98
0	39 (36–42)		6 (4–7)		37 (35–39)	
1	38 (36–42)		7 (5–7)		38 (35–40)	
2 or more	38 (24–38)		7 (4–7)		36 (36–39)	
Gender		0.30		0.56		0.72
Woman	39 (36–42)		7 (4–7)		37 (35–39)	
Man	38 (36–42)		6 (4–7)		37 (35–39)	
Race/ethnicity		0.19		0.94		0.87
White	40 (36–42)		6 (4–7)		37 (35–39)	
Hispanic	37 (36–42)		7 (4–7)		36 (35–39)	
Black or African American	37 (32–41)		7 (4–7)		38 (31–40)	
Other	38 (36–38)		6 (4–7)		37 (36–38)	
Marital status		0.13		0.11		0.22
Single	38 (36–40)		6 (4–7)		36 (34–39)	
Married	40 (36–42)		7 (5–7)		38 (35–39)	
Divorced, separated, or widowed	39 (36–42)		6 (4–7)		38 (35–40)	
Level of education		0.42		**0.004**		0.59
High school or lower	37 (35–42)		7 (7–7)		35 (35–39)	
2-year college	38 (36–42)		4 (4–7)		38 (35-40)	
4-year college	39 (36–42)		7 (4–7)		38 (35–39)	
Postgraduate degree	39 (38–41)		6 (4–7)		37 (36-39)	
Work status		**0.050**		0.46		0.95
Employed	39 (36–42)		6 (4–7)		37 (35–39)	
Unemployed	36 (36-41)		7 (5–7)		36 (35–40)	
Disabled	34 (32–40)		7 (4–7)		35 (33–42)	
Other	39 (37–42)		7 (4–7)		37 (35–39)	
Annual household income		**0.011**		0.53		0.84
Less than $15,000	36 (34–40)		7 (4–7)		35 (35–40)	
From $15,000 to $29,999	36 (35–42)		7 (5–7)		38 (35–39)	
From $30,000 to $49,999	39 (36–42)		6 (4–7)		37 (35–39)	
From $50,000 to $99,999	39 (36–42)		5 (4–7)		38 (35–39)	
More than $100,000	40 (37–42)		6 (4–7)		37 (35–39)	
Insurance status		0.29		0.76		0.15
Medicaid	39 (36–42)		7 (6–7)		31 (35–39)	
Medicare	39 (36–41)		7 (4–7)		37 (35–39)	
Private	39 (36–42)		6 (4–7)		38 (35–39)	
None or other	36 (35–40)		7 (4–7)		36 (35–39)	

Continuous variables	Spearman rank-order coefficient (ρ)	*P*-value	Spearman rank-order coefficient (ρ)	*P*-value	Spearman rank-order coefficient (ρ)	*P*-value

Age	0.15	**0.083**	0.064	0.46	0.14	0.10
NPTQ-4	−0.15	**0.083**	−0.084	0.34	0.055	0.53
PROMIS Depression	0.083	0.90	−0.0008	0.99	0.11	0.22

Continuous variables as median (interquartile range [IQR]). CEQ-6= Communication Effectiveness Questionnaire, 6-item; NPTQ-4= Negative Pain Thoughts Questionnaire, 4-item; PROMIS= Patient-Reported Outcomes Measurement Information System. All variables with *P <* 0.10 were moved to multivariable linear regression analysis. The degree of symptom exaggeration was moved to multivariable regression regardless of significance.

**Table 3:** Multivariable Poisson regression analysis of patient factors associated with communication effectiveness (CEQ-6).

Variable	Regression Coefficient (95% Confidence Interval)	Standard error	*P*-value	ΔAIC*
Symptom exaggeration				
0	*Reference Value*			
1	0.027 (−0.048 to 0.10)	0.038	0.48	7.5
2 or more	−0.21 (−0.34 to -0.078)	0.067	**0.002**	
Age	0.0016 (−0.00041 to 0.0036)	0.0010	0.12	0.44
NPTQ-4	0.0045 (−0.0025 to 0.011)	0.0036	0.21	−0.44
Work status				
Employed	*Reference Value*			
Unemployed	0.016 (−0.092 to 0.12)	0.055	0.77	−5.3
Disabled	−0.039 (−0.19 to 0.12)	0.079	0.62	
Other	0.016 (−0.058 to 0.091)	0.038	0.67	
Annual household income				
Less than $15,000	*Reference Value*			
From $15,000 to $29,999	0.0016 (−0.13 to 0.13)	0.066	0.98	−0.49
From $30,000 to $49,999	0.062 (−0.055 to 0.18)	0.060	0.30	
From $50,000 to $99,999	0.085 (−0.031 to 0.20)	0.059	0.15	
More than $100,000	0.11 (0.0089 to 0.21)	0.052	**0.033**	

**Bold** indicates statistical significance, *P* < 0.05. NPTQ-4= Negative Pain Thoughts Questionnaire. *ΔAIC = Akaike Information Criterion; AIC of the full model compared to model without each variable. Higher values indicate better fit.

**Table 4:** Bivariate analysis of symptom exaggeration associated with communication effectiveness, satisfaction, and trust in the physician, split up by hypothetical and personal scores.

	CEQ-6		Satisfaction (Guttman scale)		Trust in Physician scale	
Categorical variables	Median (IQR)	*P*-value	Mean ± SD	*P*-value	Mean ± SD	*P*-value
Symptom exaggeration (total)		0.16		0.61		0.98
0	39 (36–42)		6 (4–7)		37 (35–39)	
1	38 (36–42)		7 (5–7)		38 (35–40)	
2 or more	38 (24–38)		7 (4–7)		36 (36–39)	
Symptom exaggeration (hypothetical)		0.61		0.59		0.093
0	39 (36–42)		5 (4–7)		37 (35–39)	
1	38 (36–42)		6 (4–7)		35 (35–38)	
2 or more	38 (35–41)		6 (4–7)		38 (36–42)	
Symptom exaggeration (personal)		0.12		0.33		**0.002**
0	39 (36–42)		7 (5–7)		37 (35–39)	
1	37 (34-42)		7 (7–7)		40 (38-43)	
2 or more	10 (6–38)		7 (4–7)		14 (11–36)	

Continuous variables as median (interquartile range [IQR]). CEQ-6= Communication Effectiveness Questionnaire, 6-item.

Greater trust was associated with lower personal exaggeration alone (p=0.002) (Table 4). Multivariable analysis was not performed.

## 4 Discussion and conclusion

### 4.1 Discussion

Patients may consciously or unconsciously exaggerate symptoms in an attempt to align the clinician with their interpretation of symptoms and their weighting of the potential test and treatment options. Measures of actual or hypothetical symptom exaggeration might therefore correlate with measures of trust, communication effectiveness, and satisfaction relative to the clinician. The observation that greater communication effectiveness and trust had modest associations with symptom exaggeration suggests clinicians can benefit from listening for symptom escalation and possible exaggeration as a prompt to more effective relationship building to improve the patient’s experience of care.

There are some limitations to this study that should be taken into account. Because the sample was limited to English speaking patients in musculoskeletal clinics, additional study is merited to determine if the findings are as applicable in other settings, non-specialty care in particular. Our inclination is that these are basic human factors that will be replicated across settings. Another limitation of this study is that we piloted new symptom exaggeration questions. Our intention was to see if there was any potential relationship and then, if so, delve further in to develop formal measures of symptoms exaggeration. We studied associations and cannot determine directionality. In our opinion, directionality is not important: if a clinician notices symptoms becoming more intense or diffuse, it is likely a reflection of limited trust no matter the direction of the association.

The observed association between greater ratings of communication effectiveness and lower ratings of symptom exaggeration suggests that an increase in the intensity or diffuseness of symptoms might alert clinicians to potential opportunities for improved patient experience. These findings align with a study in two academic hospitals that found an association between less intense pain and other symptoms and nurse and patient ratings of patient-clinician communication effectiveness, along with fewer patient symptoms of anxiety and guilt.[25] The association of fewer symptoms of anxiety and guilt with better communication might reflect distress hindering communication or effective communication alleviating distress. We speculate that patients might consider reporting more severe symptoms when they don’t feel that their concerns are heard and understood. We make a similar interpretation of a California state-wide survey of 921 low income patients with breast cancer that identified an association greater perceived physician symptom awareness and fewer symptoms of nausea and depression, and lower pain intensity [26]. A qualitative study among Hispanic patients with breast cancer noted that patients are often unhappy with clinician responses to their symptoms [27]. Additional quantitative and qualitative investigation can investigate the degree to which this dissatisfaction might motivate symptom exaggeration in an attempt elicit a more satisfying response.

The observation that greater ratings of trust in physician were associated with lower personal symptom exaggeration scores suggests that exaggeration may signal limited trust. Although other studies have not assessed symptom exaggeration directly, our findings are in line with evidence that a better patient experience, trust in particular, is associated with fewer and less intense symptoms. For instance, a survey study of 119 patients with different types of cancer (breast, cervix, intestine, and prostate) found that lower trust was associated with more intense symptoms, greater emotional distress, and greater incapability [28]. It is possible that people report more symptoms to signal “I’m not sure I you trust you,” a possibility that merits additional investigation. A meta-analysis found that greater trust was associated with fewer and less intense symptoms and greater satisfaction with care [29]. Due in part to social stigma regarding mental health, it can be more acceptable to express distress in terms of physical symptoms (somatization) [30]. It is therefore plausible that, in the context of a trusting patient-physician relationship that facilitates more comfortable discussion of delicate topics such as potential misinterpretation of symptoms and feelings of despair and worry, we might observe less intense and less diffuse symptoms. The authors interpret many of our day-to-day interactions with patients along these lines.

The observed association between higher satisfaction and lower education is similar to a cross-sectional study of 650 patients discharged from medical and surgical wards of 4 acute care general hospitals that found that patients with very limited education had higher satisfaction scores [31]. A survey study of 500 people discharged from a military teaching hospital also found that lower education correlated with higher satisfaction [32]. A cross-sectional study measuring patients and clinicians’ perceptions and patient satisfaction of 134 male patients and 12 physicians receiving care at a diabetes outpatient clinic found that patients with lower education were most satisfied [33]. Another cross-sectional study addressing colonoscopy patients’ satisfaction 72 hours after the procedure found that higher education was associated with dissatisfaction with care [34]. A study of patient satisfaction among 200 patients admitted to various hospital wards found that lower education was associated with higher satisfaction [35]. A meta-analysis measuring the association of sociodemographics and satisfaction found that greater satisfaction was consistently associated with lower education [36]. There are a few outliers, including a survey-based cross sectional study addressing determinants of patient satisfaction among 237 consecutive patients seeking care for upper respiratory infections in an outpatient practice, which found no statistical relationship between education and patient satisfaction [37].

### 4.2 Innovation

We have identified a potential useful nuance in the concept of symptom amplification. People may intentionally represent their symptoms as more intense, diffuse, or impactful as a means signaling that they don’t feel heard and understood. Going forward, clinicians can see symptom amplification as a signal of unhelpful thinking and feelings of distress regarding symptoms, but also as a cue that they are no communicating effectively. Clinicians can be taught to regard symptom amplification as a signal that their communication strategies have not established trust and understanding and should be adjusted.

### 4.3 Conclusion

Our results suggest that symptom descriptions that seem more intense or diffuse than expected, may indicate opportunities for more effective communication and trust, particularly if the symptoms increase during a specialty care visit. Symptom exaggeration may be a tool patients sometimes use to convey deficiencies in the patient-specialist relationship. Future studies can attempt to confirm this association and then further investigate strategies for identifying symptom exaggeration and then implementing targeted strategies to improve patient experience.

## Disclosure

This research did not receive any specific grant from funding agencies in the public, commercial, or not-for-profit sectors. All authors indicated that they had no conflicts of interest.

## Ethical approval

Approval was obtained by the Institutional Review Board before conducting this study (ID: 2020−04−0108).

## Statement of human and animal rights

All procedures in this study were conducted in accordance with the Institutional Review Board approved protocols.

## Informed consent

Verbal informed consent was obtained from the patients for their anonymized information to be published in this article prior to supplying them with questionnaire.

## Declaration of Competing Interest

The authors declare that they have no known competing financial interests or personal relationships that could have appeared to influence the work reported in this paper.

## Figures and Tables

**Table:** 

Variable	Regression coefficient (95% confidence interval)	Standard error	*P*-value	ΔAIC*
Symptom exaggeration				
0	*Reference value*			
1	0.052 (−0.13 to 0.23)	0.091	0.57	−3.7
2 or more	0.0016 (−0.29 to 0.29)	0.15	0.99	
Level of education				
High school or lower	*Reference value*			
2-year college	−0.21 (−0.45 to 0.033)	0.12	0.091	−2.5
4-year college	−0.083 (−0.27 to 0.11)	0.097	0.39	
Postgraduate degree	−0.15 (−0.34 to 0.052)	0.10	0.15	

**Bold** indicates statistical significance, *P* < 0.05. NPTQ-4= Negative Pain Thoughts Questionnaire. *ΔAIC = Akaike Information Criterion; AIC of the full model compared to model without each variable. Higher values indicate better fit.

**Figure:**
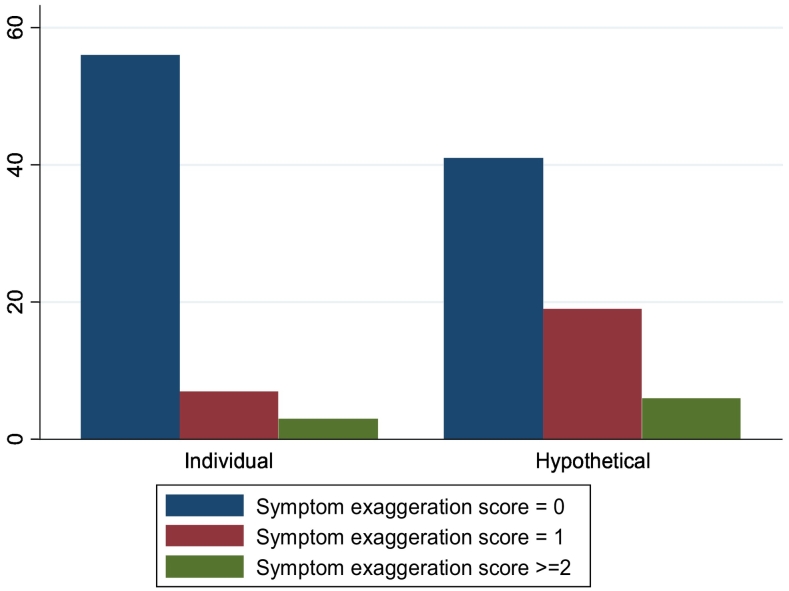


**Figure:**
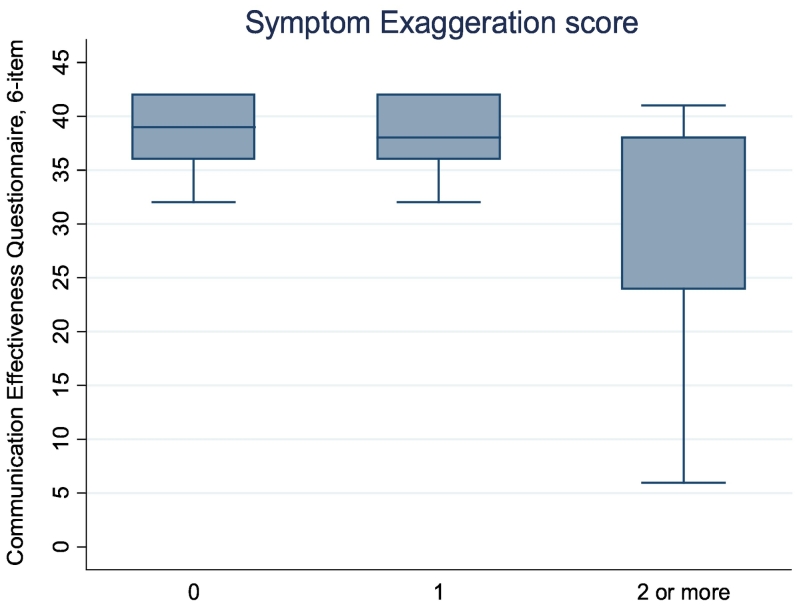


**Figure:**